# Surface scanned 3D designed customized chinstrap for the treatment of intraoral dehiscence in polytraumatized patient. Technical note

**DOI:** 10.4317/jced.61339

**Published:** 2024-02-01

**Authors:** Íñigo Aragón-Niño, José-Luis del Castillo-Pardo-de Vera, Alessandro Gutiérrez-Venturini, Marta-María Pampín-Martínez, Clara López-Martínez, José-Luis Cebrián-Carretero

**Affiliations:** 1 Medical Resident. Oral and Maxillofacial Surgery Department. La Paz University Hospital. Madrid, Spain; 2 Physician attending / Faculty. Oral and Maxillofacial Surgery Department. La Paz University Hospital. Madrid, Spain; 3 Bioengineer. Oral and Maxillofacial Surgery Department. La Paz University Hospital. Madrid, Spain; 4 Head of the Department. Oral and Maxillofacial Surgery Department. La Paz University Hospital. Madrid, Spain

## Abstract

The advent of 3D surgical technology has revolutionized personalized medicine, enabling the development of tailored solutions for individual patients. This technical note presents the application of 3D technology in designing a customized chin guard using flexible 3D resin. The process involves surface scanning the lower facial region of a polytraumatized patient with a structured-light surface 3D scanner, generating a detailed point cloud. The acquired data undergoes meticulous processing within an specific professional software, including erasing unwanted portions, aligning frames, and mesh consolidation. Subsequently, the mesh is exported as an STL file and further refined using a 3D mesh management software. A customized chin support is designed for the specific patient’s needs, exported in STL format, and 3D printed using a stereolithography (SLA) printer with Flexible 80A resin. Post-printing procedures involve washing and curing to ensure biocompatibility and optimal mechanical characteristics. The resultant customized chin guard, attached to elastic support straps, offers a precise fit to the patient’s anatomy, enhancing comfort and allowing for extended wear. This innovative approach addresses the challenge of surgical intraoral wound dehiscence in a polytraumatized patient, showcasing the potential of 3D technology in personalized medical solutions for complex cases.

** Key words:**Surface scanner, 3D surgery, customized surgery, chinstrap.

## Introduction

3D surgery has meant an advance in the quality of care, allowing the creation of customized solutions and devices for the patient, raising personalized medicine to the highest level (1,2). This technical note describes the use of this technology applied to the creation of a customized chin guard printed in flexible 3D resin whose design has been obtained after scanning the patient’s chin region with a surface scanner.

## Case Report

This article reports on the case of a polytraumatized patient after an occupational accident with a radial saw that caused severe facial and cervical injuries and maxillary and mandibular fractures.

After stabilization of the patient, he underwent surgery for reduction of the facial fractures and osteosynthesis fixation treatment. Of special relevance was the injury at mandibular level where he suffered a comminuted fracture with great avulsion of soft tissues.

The postoperative period evolved favorably, with only a intraoral dehiscence in the third quadrant at the vestibular region, caused by the severe soft-tissue involvement in the accident. To solve this problem, we decided to design a customized chin strap that would provide specific support at the level of the dehiscence and that could compress in a controlled level every anatomical part of the patient’s altered soft tissue in that area of the chin. The objective was the design of a customized device adapted to the patient’s soft tissues to support this region and helping to reduce a surgical intraoral wound dehiscence.

In this case we needed a solution that would allow us to design and create an orthosis completely customized to the soft surface of the patient’s skin. The first step was to be able to analyze the anatomy at that level. The use of surface scanning has proven especially useful and accurate in the lower third of the face (3,4).

**Figure 1:**
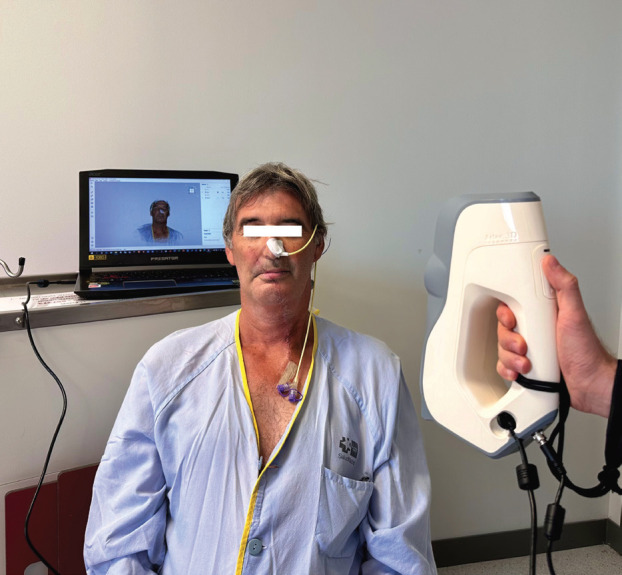
Surface scanning process on the patient.

Using a structured-light surface 3D scanner (Artec Eva, Artec Group, Luxembourg), the lower third of the face was surface-scanned in the first step. With this scanner, surfaces can be captured with a 3D resolution of up to 0.2 mm resolution and a reconstruction rate of 16 fps while additionally preserving the texture of the model, (Fig. 1).

**Figure 2:**
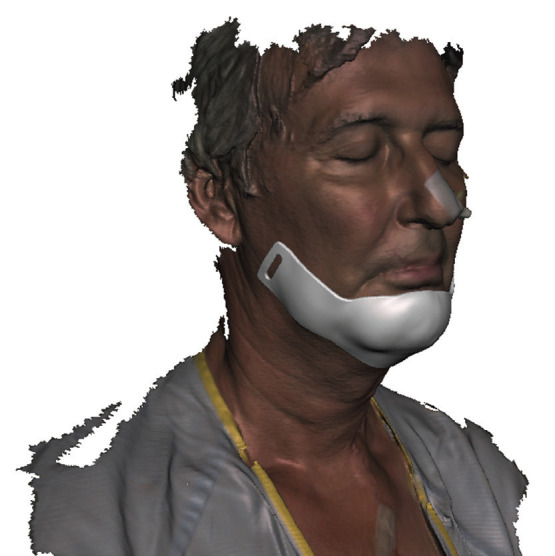
3D design of the chin rest on the patient’s scan with Meshmixer software.

The acquired point cloud data underwent a series of processing steps within the Artec Studio 17 Professional software (Artec Group, Luxembourg). First, the erasing tool was employed to enable manual removal of unwanted portions in the point cloud data, such as the background. Subsequently, the registration tool assessed and aligned all frames obtained during the scanning procedure. By utilizing the outlier removal tool, extraneous points surrounding the subject were eliminated, reducing noise. Following this, a consolidation of all the frames was executed to generate a unified mesh. Finally, the mesh received texture from the original scan, (Fig. 2).

In the next step, the mesh that was acquired was exported as an STL file and then underwent processing with the modeling software Meshmixer (Autodesk Inc., San Rafael, CA, USA).

With this software, a custom chin support is designed for the specific area where support is needed. For the design of the chin rest, a combined structure with support with elastic bands at cranial level was considered. A buckle system was designed for the bands with the customized orthosis, as it was found to be more reliable and resistant than other alternatives. An alternative model without a buckle and with support directly on a standard elastic chin strap was also designed but was finally discarded because it did not allow control of the degree of pressure and was uncomforTable for the patient.

This design was exported as a 3D object in STL format and then printed in a stereolithography (SLA) 3D printer (Form 2, Formlabs, Somerville, MA, USA) with Flexible 80A resin (Formlabs) at a 0.1 mm priting resolution.

**Figure 3:**
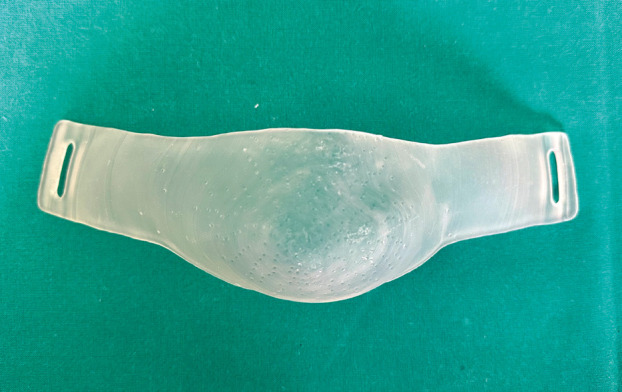
Customized printed chin strap.

Following the printing process, the models were taken off the build platform and immersed in a Form Wash (Formlabs) containing 99% isopropyl alcohol for a 20 minute duration. This step was carried out to cleanse the components and eliminate any remaining liquid resin. Subsequently, they underwent a 10 minute post-curing process at 60ºC in a Form Cure (Formlabs) to attain both biocompatibility and optimal mechanical characteristics, (Fig. 3).

**Figure 4:**
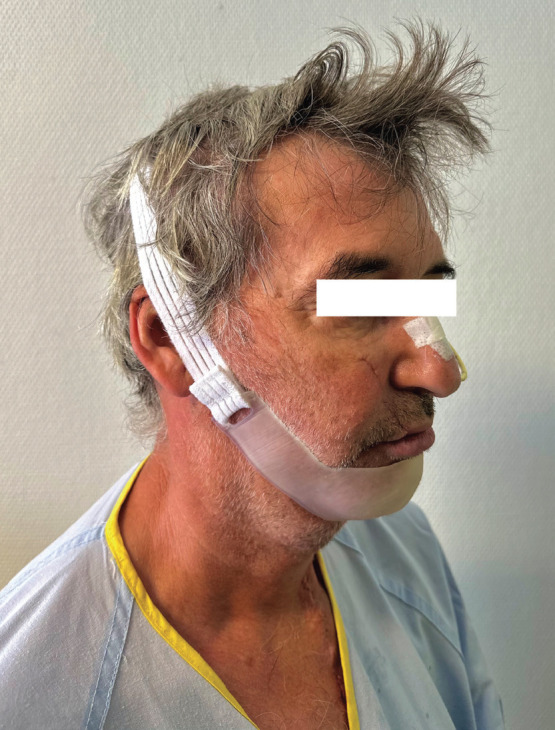
Customized chin guard being used by the patient.

Finally the model was tested with the patient with excellent acceptance on his part due to its comfort and perfect adaptation to his soft tissue contour, which avoided areas of increased unwanted pressure, skin folds and skin abrasions. The patient used the chin guard for 2 weeks continuously 24 hours a day and another two weeks intermittently, which resulted in a satisfactory resolution of the intraoral dehiscence, (Fig. 4).

Consent was obtained from the patient for the publication of the case progress records.

## Discussion

Advances in technology and specifically in 3D surgery are allowing the design and creation of fully customized solutions to the patient and the specific case we have to treat(5). This gives us specific advantages that determine a better quality of care for our patients. In the case described, the patient’s poor tolerance to generic chin straps led to poor adherence to treatment and insufficient compliance. The possibility of providing him with a specific solution adapted to his anatomy led to a radical improvement in adherence. In addition, the specific design to his facial anatomy allowed us to create a surface on which the pressure was distributed in a uniform and controlled manner without damaging the soft tissues that were already very affected by the trauma.
